# Banxia-Shengjiang drug pair inhibits gastric cancer development and progression by improving body immunity

**DOI:** 10.1097/MD.0000000000036303

**Published:** 2024-03-08

**Authors:** Yating Yang, Ling Yuan, Wenjing Liu, Doudou Lu, Fandi Meng, Yi Yang, Ziying Zhou, Ping Ma, Yi Nan

**Affiliations:** a Traditional Chinese Medicine College, Ningxia Medical University, Yinchuan, Ningxia, China; b College of Pharmacy, Ningxia Medical University, Yinchuan, Ningxia, China; c Key Laboratory of Hui Ethnic Medicine Modernization of Ministry of Education, Ningxia Medical University, Yinchuan, Ningxia, China; d School of Clinical Medicine, Ningxia Medical University, Yinchuan, Ningxia, China; e Pharmacy Department, General Hospital of Ningxia Medical University, Yinchuan, Ningxia, China.

**Keywords:** Banxia-Shengjiang, bioinformatics, drug pair, gastric cancer, network pharmacology

## Abstract

To investigate the mechanism of action of Banxia-Shengjiang drug pair on the inhibition of gastric cancer (GC) using network pharmacology and bioinformatics techniques. The action targets of the Banxia (*Pinellia ternata (Thunb.) Makino*) -Shengjiang (*Zingiber officinale Roscoe*) drug pair obtained from the TCMSP database were intersected with differentially expressed genes (DEGs) and GC-related genes, and the intersected genes were analyzed for pathway enrichment to identify the signaling pathways and core target genes. Subsequently, the core target genes were analyzed for clinical relevance gene mutation analysis, methylation analysis, immune infiltration analysis and immune cell analysis. Finally, by constructing the PPI network of hub genes and corresponding active ingredients, the key active ingredients of the Banxia-Shengjiang drug pair were screened for molecular docking with the hub genes. In this study, a total of 557 target genes of Banxia-Shengjiang pairs, 7754 GC-related genes and 1799 DEGs in GC were screened. Five hub genes were screened, which were PTGS2, MMP9, PPARG, MMP2, and CXCR4. The pathway enrichment analyses showed that the intersecting genes were associated with RAS/MAPK signaling pathway. In addition, the clinical correlation analysis showed that hub genes were differentially expressed in GC and was closely associated with immune infiltration and immunotherapy. The results of single nucleotide variation (SNV) and copy number variation (CNV) indicated that mutations in the hub genes were associated with the survival of gastric cancer patients. Finally, the PPI network and molecular docking results showed that PTGS2 and MMP9 were potentially important targets for the inhibition of GC by Banxia-Shengjiang drug pair, while cavidine was an important active ingredient for the inhibition of GC by Banxia-Shengjiang drug pair. Banxia-Shengjiang drug pair may regulate the immune function and inhibit GC by modulating the expression of core target genes such as RAS/MAPK signaling pathway, PTGS2 and MMP9.

## 1. Introduction

Gastric cancer (GC) is a common malignant tumor of the gastrointestinal tract, characterized by high incidence, high mortality, poor prognosis, easy recurrence and metastasis.^[1,2]^ According to GLOBOCAN statistics, in 2020, there were an estimated 19.3 million new cases and 10 million cancer deaths worldwide, with gastrointestinal cancers being one of the leading causes of death.^[3]^ Currently, GC is mainly treated by surgical resection, radiotherapy, chemotherapy and targeted therapy,^[4,5]^ but for metastatic gastric cancer patients, surgery is difficult to eradicate, and chemotherapeutic drugs not only have strong toxicity and side effects, but also lead to drug resistance in long-term use,^[6,7]^ therefore, the development of new drugs with low side effects and anticancer effects is of great clinical significance. Studies have shown that some natural Chinese medicinal ingredients show unique advantages in anticancer, which can not only alleviate the clinical problems of strong side effects of chemotherapeutic drugs and drug resistance, but also enhance the patient own immune system, play an anti-tumor activity, thus improving the patient quality of life.

Banxia-Shengjiang drug pair is the core pair of many compound prescriptions for digestive diseases, such as Xiao-Ban-Xia Decoction, Shengjiang Banxia Decoction, Banxia Xiexin Decoction, etc, which are all from the “*Treatise on Typhoid Miscellaneous Diseases*,” with a history of medicinal use of more than 2000 years, and have excellent therapeutic efficacy in treating digestive disorders.^[8]^ Banxia-Shengjiang drug pair is a commonly used combination of Traditional Chinese Medicine (TCM), and practical research has found that Shengjiang (*Zingiber officinale Roscoe*) can not only reduce the toxicity, but also enhance the efficacy of Banxia (*Pinellia ternata (Thunb.) Makino*).^[9]^ Therefore, the combination of the 2 has an inherent advantage. Banxia-Shengjiang drug pair should be used clinically to treat a variety of difficult and complicated diseases, including anti-tumor research, the exploration of its active ingredients suggests that a variety of components in Banxia-Shengjiang drug pair have anti-tumor effects.^[10]^ Baicalein, as an important active ingredient of Banxia,^[11]^ could inhibit the proliferation and EMT process of GC cells, and its mechanism was related to the regulatory cycle and the expression level of EMT-related proteins.^[12]^ Meanwhile, Shengjiang extract and 6-gingerol inhibited GC cell proliferation and induced apoptosis and G1-phase blockage of the cell cycle,^[13]^ which provided a new basis for the treatment of GC as a natural medicine.^[14,15]^

The exertion of Banxia-Shengjiang drug pair action is also reflected in numerous formulas, such as Banxia Xiexin Decoction and Xiao-Ban-Xia Decoction. Guoxiu Zu et al^[16]^ found that Banxia Xiexin Decoction could inhibit the proliferation and accelerate the apoptosis of gastric mucosal epithelial cells and intrinsic cells by inhibiting the aberrant activation of key factors of the MAPK/ERK signaling pathway, thus preventing the occurrence of carcinoma. Ningning Liu et al^[17]^ found that Banxia Xiexin Decoction could inhibit GC cells invasion and metastasis by inhibiting signaling pathways such as VEGF, MMP-2, MMP-7, MMP-9 and PI3K/AKT. Lei Xia et al^[18]^ found that Banxia-Shengjiang drug pair had a synergistic promotional effect on the proliferation and apoptosis-inducing process of ovarian cancer SKOV3 cells, and the mechanism might be related to the induction of ROS generation, iron death, and inhibition of the PI3K/AKT/mTOR signaling pathway. Not only that, Jing Leng et al^[19]^ found that Xiao-Ban-Xia Decoction had a better prevention and treatment effect on patients with chemotherapeutic nausea and vomiting in GC, which could significantly improve the adverse symptoms of patients, and had a good prospect of clinical application.

Immunotherapy, as a current research hotspot and emerging treatment method in the field of tumor therapy, can improve the survival rate of patients with middle- and late-stage GC. Tumor immunotherapy has the advantages of being specific and efficient, and freeing the organism from injurious treatments.^[20]^ Different in nature from traditional treatments such as surgery, targeting, radiotherapy, etc, immunotherapy does not kill cancer cells directly, but mobilizes immune cells in the body that can recognize tumors, improves the immune system in the body to fight, and relies on them to indirectly kill and control cancer, with little side effects, safe and effective. It can be seen that immunotherapy and TCM treatment have similar roles in anti-tumor, with small side effects, safe and effective natural medicines have attracted people attention, and the study of TCM-related anti-tumor mechanisms and medicines has become a hotspot of research.^[21]^ For example, Banxia Xiexin Decoction and its dismantling formula have potential targets and mechanisms of action to improve intestinal immunity in inflammatory bowel disease.^[22]^ Xiao-Ban-Xia Decoction can also improve the microenvironment of immunotherapy through ERK1/2 signaling pathway to improve xenophagy in rats.^[23]^ As TCM research enters the molecular era, the utilization of in vivo and ex vivo experimental studies through the establishment of effective experimental standards and evaluation criteria will advance the understanding of the therapeutic mechanisms of TCM and make it an alternative choice for cancer treatment. Current studies have shown that TCM has significant efficacy in inducing apoptosis in GC cells, improving body immunity, reducing adverse reactions caused by chemotherapy, and enhancing its antitumor effects in combination with chemotherapy.^[24]^

**Figure 1.**
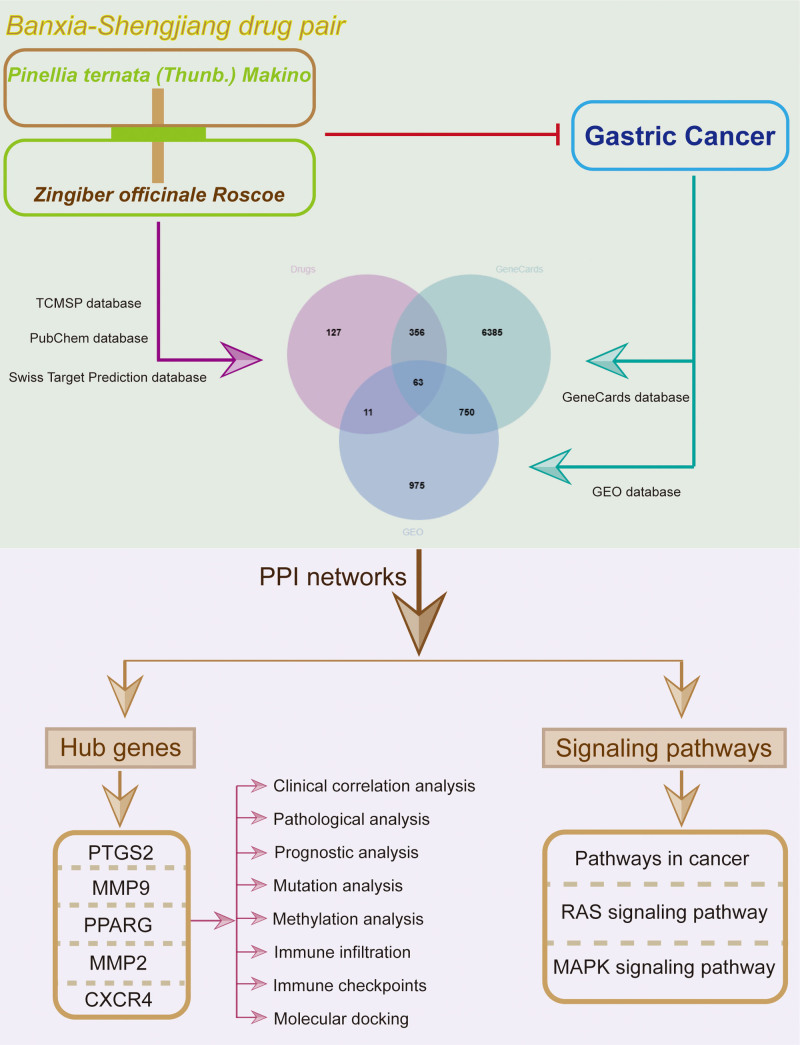
Flow chart of the study.

Currently, the combination of bioinformatics and network pharmacology has been widely used in the research of TCM.^[25]^ Based on this, established a drug-target-disease network to explore the core targets, biological functions, pathways, and mechanisms of Banxia-Shengjiang drug pair for the treatment of GC. In this study, we found that Banxia-Shengjiang drug pair may inhibit the activity of GC through the RAS/MAPK signaling pathway. We also found that Banxia-Shengjiang drug pair is closely related to immunotherapy, and may exert anti-GC activity by regulating the function of immune cells and improving body immunity. The flowchart is shown in Figure 1.

## 2. Materials and methods

### 2.1. Access to drug target genes

The TCMSP database 2.3 (https://old.tcmsp-e.com/tcmsp.php) was used to search for the active ingredients using the keywords “Banxia” and “Shengjiang,” respectively.^[26]^ The search results were screened for oral bioavailability (OB) ≥ 30% and drug-like properties (DL) ≥ 0.18,^[27]^ and the eligible active ingredients were obtained. Next, the active ingredients were entered into the PubChem database (https://pubchem.ncbi.nlm.nih.gov) to obtain the structural formula of SMILES,^[28]^ and the Swiss Target Prediction database (http://www.swisstargetprediction.ch) was utilized to prediction of its target, probability > 0 was used as the screening condition. The predicted active ingredients and targets were plotted on the “Ingredient-Target Network Diagram” in Cytoscape 3.9.1 software.

### 2.2. Access to GC-related genes and DEGs

GC-related genes were obtained from GeneCards database (https://www.genecards.org/),^[29]^ using “Gastric cancer” as search term and Relevance score > median as filtering criteria. The DEGs of GC genes was obtained from the GEO database (https://www.ncbi.nlm.nih.gov/geo),^[30]^ and the original GeneChip data number GSE118916 was downloaded, and then analyzed online by GEO2R,^[31]^ and DEGs were screened by the criteria of |LogFC|>1 with *P* < .05 (Supplemental Digital Content 1 and 2, http://links.lww.com/MD/L710).

### 2.3. Access to intersecting genes

The intersection of the drug-predicted target genes was taken with the up-regulated and down-regulated genes in the DEGs. Their Venn diagram were plotted using W bioinformatics (http://www.bioinformatics.com.cn/) and GraphPad Prism 8.0 plotted the volcano plot. After that, the Venn diagram of the intersection between drug- GC -related genes-DEGs was plotted to obtain the intersecting genes. In addition, a heatmap of the intersecting genes were plotted. Finally, the intersecting targets were imported into the W bioinformatics for PCA processing and analysis to evaluate the intergroup and intragroup differences.^[32]^

### 2.4. Construction and functional analysis of intersecting gene PPI networks

The intersecting genes between drugs, GC-related genes and DEGs were imported into the STRING database (https://string-db.org) for protein-protein interaction (PPI) analysis.^[33]^ The PPI network of intersecting genes was constructed according to the Degree value, and the core targets were presented in the form of bar graphs. The correlations between the core targets were explored using the GEPIA 2.0 database (http://gepia2.cancer-pku.cn),^[34]^ and the results were presented by drawing a heatmap of intragroup differences using the W bioinformatics. For functional analysis, the intersecting genes were imported into the DAVID database (https://david.ncifcrf.gov),^[35]^ and the results were analyzed by pathway enrichment using the Sangerbox 3.0 database (http://sangerbox.com/home.html).^[36]^ Finally, we explored the “Pathway Enrichment” module in the CAMOIP database (https://www.camoip.net) and selected the “TCGA-Cohort” dataset.^[37]^ In order to analyze the correlation between the core targets and the key pathways and the biological processes (BP).^[38]^

### 2.5. Clinical correlation analysis of the hub genes

Firstly, we analyzed the expression differences of hub genes using the “gene expression differential analysis” module in Sangerbox 3.0 database. Then we analyzed the copy number change, protein expression and stage of hub genes using the “Expression DIY” module of GEPIA2.0 database. The correlation between hub genes and GC subtypes was investigated in the “Expression” module of the GSCA database (http://bioinfo.life.hust.edu.cn/GSCA/#/).^[39]^

### 2.6. Pathologic findings and prognostic analysis of the hub genes

Immunohistochemical images were obtained using the Human Protein Atlas database (https://www.proteinatlas.org) to analyze the hub genes in the tissues, and immunofluorescence images were obtained to analyze their localization in the tumor tissues.^[40]^ Secondly, the relationship between the hub genes in GC and the overall survival (OS) of patients was analyzed by plotting the survival curve using the “gastric cancer” module in the Kaplan–Meier Plotter database (http://kmplot.com).^[41]^ A cox proportional-hazards model (COX) was also constructed in CAMOIP to investigate the correlation between prognostic survival and clinical factors.^[42]^

### 2.7. Mutations in the hub genes

We first analyzed the sites and types of single nucleotide variation (SNV) mutations in the hub genes using the “Mutation” module in the GSCA database,^[43]^ and presented the frequency of deleterious mutations as a heat map percentage. Further exploring the effects of copy number variation (CNV),^[44]^ mutation data are presented in bubble plots. In addition, we explored the top 20 Driver Mutation genes associated with hub genes in the CAMOIP database,^[45]^ including Oncogene, TSG, and Unknown classified genes. The “Immunogenicity” module was also used to analyze the MSI, TMB and Neoantigene of the tumor.

### 2.8. Methylation of hub genes

The UALCAN database (https://ualcan.path.uab.edu) can be used to explore the relationship between gene methylation and disease by plotting methylation expression box plots in the “TCGA” module.^[46]^ The correlation between hub genes methylation and cytotoxic T-lymphocytes (CTL) was also obtained from the TIDE database (http://tide.dfci.harvard.edu/login),^[47]^ and the prognostic survival of GC was analyzed in the hypermethylated and hypomethylated groups.

### 2.9. Immune infiltration and immune checkpoints

Firstly, we plotted the immune infiltration analysis using W bioinformatics to observe the expression of DEGs in immune cells.^[48]^ After that, we plotted the Stromal Score, Immune Score and EATIMATE Score scatter plots of hub genes and GC using the “Immune Infiltration Analysis (Estimate)” module of Sangerbox.^[49]^ In addition, we selected “no treatment” in TISCH2 database (http://tisch.comp-genomics.org/home) for single-cell annotated profiles in the final dataset.^[50]^ Finally, based on the results of single-cell annotation profiles,^[51]^ we further analyzed the correlation between macrophages, monocytes, CD8T cells and tumor-associated fibroblasts and hub genes in GC using the TIMER 2.0 database (http://timer.cistrome.org).

### 2.10. Molecular docking validation

The “drug-ingredient-target” network map screened the top 5 key ingredients based on the Degree value, Cavidine, (3S,6S)-3-(benzyl)-6-(4-hydroxybenzyl) piperazine-2,5-quinone, baicalein derived from Banxia, and 6-methylgingediacetate2, Dihydrocapsaicin derived from Shengjiang. PTGS2, MMP9, PPARG, MMP2 and CXCR4 were molecularly docked to these 5 key components. Obtain the 2D and 3D structures of the key components from PubChem database, and download the “pdb” format of the core target from the PDB database (http://www.rcsb.org).^[52]^ Then run AutoDock Vina tool for molecular docking. The binding strength and activity were evaluated according to the Docking Score, and the docking results were optimized and mapped with the help of PyMOL software.

## 3. Results

### 3.1. Access to active ingredients and targets of drugs

**Figure 2.**
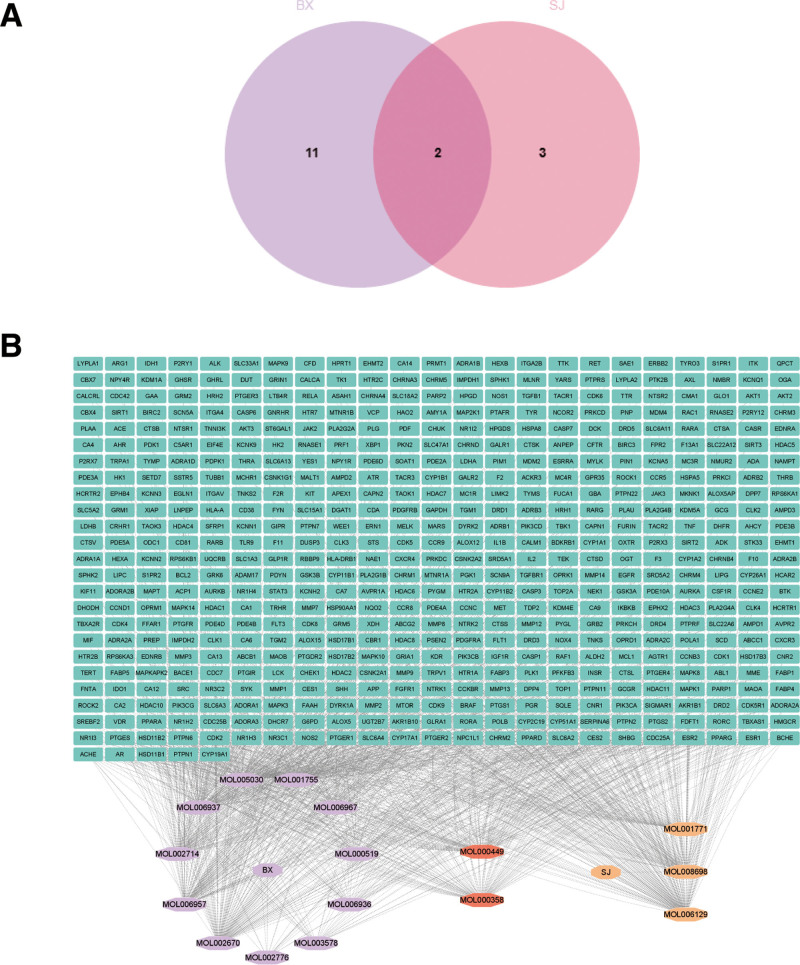
Access to drug active ingredients and targets. (A) Venn diagram of the active components of Banxia-Shengjiang. (B) Network diagram of “drug-active ingredient-target.” The square icon represents the target of the drug, and the diamond represents the active ingredient of each Traditional Chinese Medicine, among which the orange-red diamond icons “MOL000449” and “MOL000358” are the common ingredients of Banxia-Shengjiang.

Screening of the active ingredients of the drugs from TCMSP database obtained 13 active ingredients of Banxia and 5 active ingredients of Shengjiang, of which MOL000358 and MOL000449 were shared components (Table 1). Obtained 462 active ingredient targets for Banxia and 258 active ingredient targets for Shengjiang. A total of 16 active ingredients and 557 active ingredient targets were obtained after merging and de-duplication. A Venn diagram was drawn to show the intersection of active ingredients of the 2 drugs (Fig. 2A). And a network diagram of “drug-active ingredient-target” was constructed (Fig. 2B).

**Table 1:** These active phytochemicals of Banxia-Shengjiang drug pair.

Herb Name	Mol ID	Molecule Name	OB (%)	DL
Banxia (*Pinellia ternata (Thunb.) Makino*)	MOL001755	24-Ethylcholest-4-en-3-one	36.08	0.76
	MOL002670	Cavidine	35.64	0.81
	MOL002714	baicalein	33.52	0.21
	MOL002776	Baicalin	40.12	0.75
	MOL005030	gondoic acid	30.7	0.2
	MOL000519	coniferin	31.11	0.32
	MOL006936	10,13-eicosadienoic	39.99	0.2
	MOL006937	12,13-epoxy-9-hydroxynonadeca-7,10-dienoic acid	42.15	0.24
	MOL006957	(3S,6S)-3-(benzyl)-6-(4-hydroxybenzyl)piperazine-2,5-quinone	46.89	0.27
	MOL003578	Cycloartenol	38.69	0.78
	MOL006967	beta-D-Ribofuranoside, xanthine-9	44.72	0.21
Shengjiang (*Zingiber officinale Roscoe*)	MOL006129	6-methylgingediacetate2	48.73	0.32
	MOL001771	poriferast-5-en-3beta-ol	36.91	0.75
	MOL008698	Dihydrocapsaicin	47.07	0.19
Banxia-Shengjiang drug pair	MOL000358	beta-sitosterol	36.91	0.75
	MOL000449	Stigmasterol	43.83	0.76

DL = drug-like properties, OB = oral bioavailability.

### 3.2. Prediction of potential targets for drug treatment

**Figure 3.**
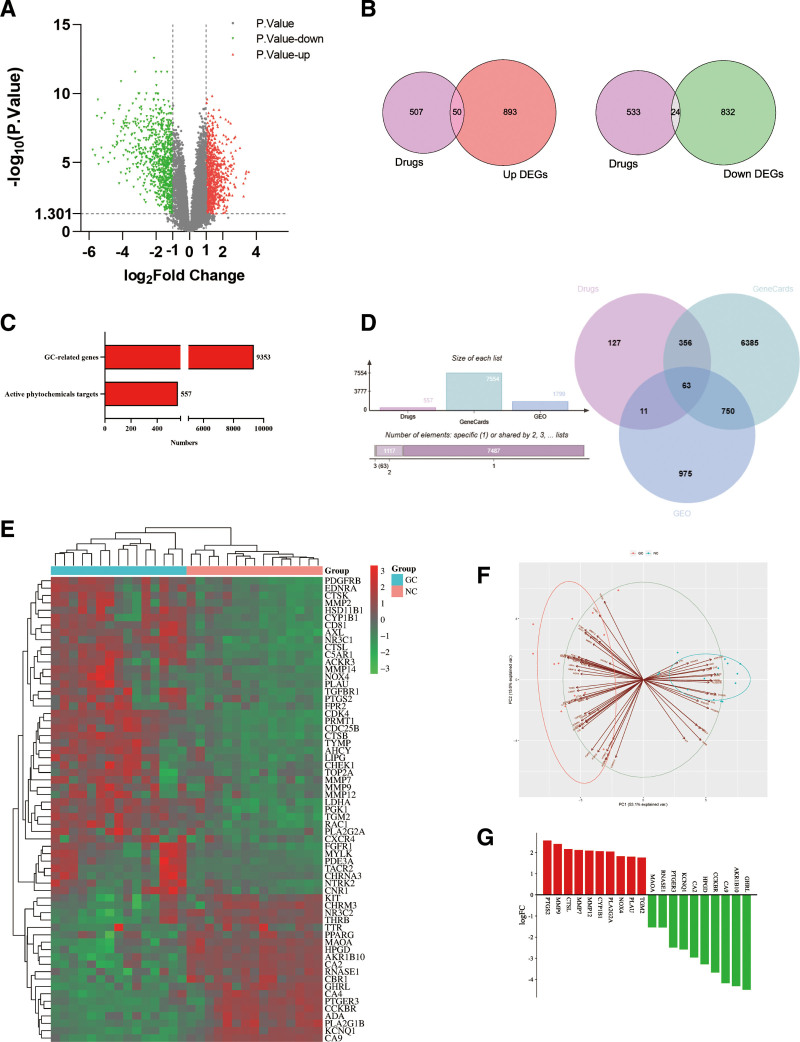
Access to gastric cancer-related genes and DEGs. (A) Volcano plot analysis of differentially expressed genes. Red represents up-regulated genes, green represents down-regulated genes. (B) Venn diagram of drug targets with up- and down-regulated genes, respectively. (C) Statistical chart of the number of disease-related targets and drug targets. (D) Intersection Venn diagram between drug-GC-related genes-DEGs. (E) Heat map of intersecting genes. (F) PCA analysis. (G) The top 10 up-regulated genes and the top 10 down-regulated genes with the most significant differentially expressed genes.

The GEO database was utilized to screen and obtain 1799 DEGs. Volcano plot analysis of the DEGs was performed using GraphPadPrism 8.0 (Fig. 3A). The Venn diagram showed that the drug targets were taken to 50 intersections with up-regulated genes, and 24 intersected genes with down-regulated genes (Fig. 3B). 7554 GC-related target genes were obtained from the GeneCards. A total of 9353 potential targets for the treatment of GC (Fig. 3C). The intersection of the 3 was taken to obtain 63 intersecting targets (Fig. 3D). PCA analysis of the GEO data showed that the samples in the normal and GC groups were clearly separated, which indicated that the GEO data were reliable (Fig. 3F). The results of the heat map of intersecting genes showed that about 1/3 of the intersecting genes showed an increasing trend of expression in gastric cancer, and 2/3 showed an obvious decreasing trend (Fig. 3E). The top 10 up-regulated and the top 10 down-regulated genes with the most significant expression differences were found (Fig. 3G).

### 3.3. Construction of intersecting gene PPI networks

**Figure 4.**
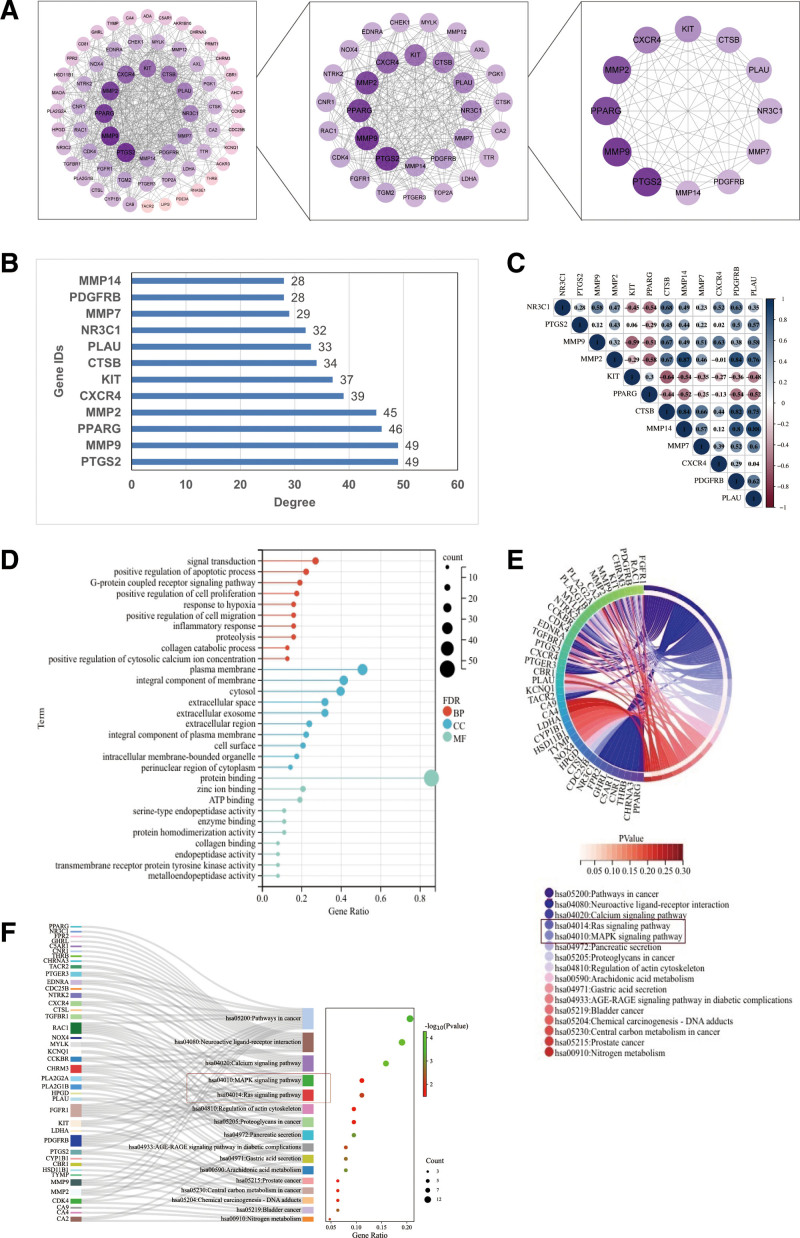
PPI and enrichment analysis. (A) PPI networks of intersecting genes were screened twice with Degree ≥ 21 and ≥ 28 for final core targets. (B) Intersecting genes of the first 12 with Degree value ≥ 28. (C) The first 12 genes were analyzed for correlation. (D) GO analysis. (E) KEGG pathway enrichment analysis. (F) Sankey dot pathway enrichment.

Two screenings were conducted on PPI networks with Degree ≥ 21 and ≥ 28 to obtain the final core targets (Fig. 4A). The top 12 intersecting genes with Degree value ≥ 28 were, in order, PTGS2, MMP9, PPARG, MMP2, CXCR4, KIT, CTSB, PLAU, NR3C1, MMP7, PDGFRB, and MMP14 (Fig. 4B). These 12 genes were analyzed by correlation analysis (Fig. 4C), and they might work together to play a therapeutic role in GC.

### 3.4. GO, KEGG and GSEA analysis

**Figure 5.**
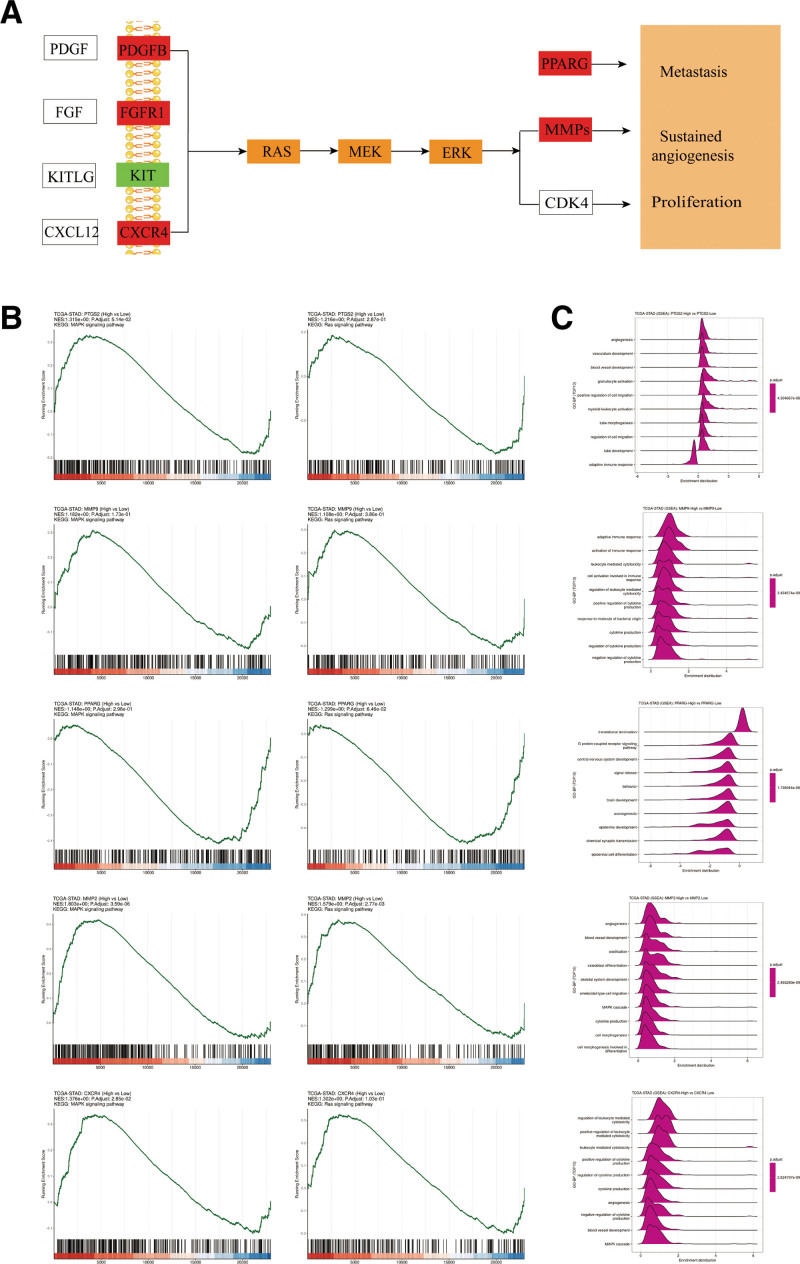
Pathway analysis. (A) Schematic diagram of key pathways. (B) GSEA analysis of RAS signaling pathway, MAPK signaling pathway in KEGG pathway by hub genes. (C) Analysis of biological processes by hub genes.

GO analysis showed that on BP, the intersecting genes were mainly associated with signal transduction, positive regulation of apoptotic process and G-protein coupled receptor signaling pathway. In terms of cellular components, were mainly associated with plasma membrane, integral component of membrane, cytosol and extracellular space. And in terms of molecular function were mainly associated with protein binding, zinc ion binding and ATP binding (Fig. 4D). KEGG analysis showed that the intersected genes were mainly related to Pathways in cancer, RAS signaling pathway and MAPK signaling pathway (Fig. 4E). Sankey dot pathway enrichment also showed that multiple targets were enriched in the pathway (Fig. 4F). According to the GSEA analysis and BP of RAS signaling pathway,^[53]^ MAPK signaling pathway in KEGG pathway by hub genes, it was found that the core genes had a role in the BP of GC treatment and the signaling pathways studied (Fig. 5).

### 3.5. Clinical correlation analysis of the hub genes

**Figure 6.**
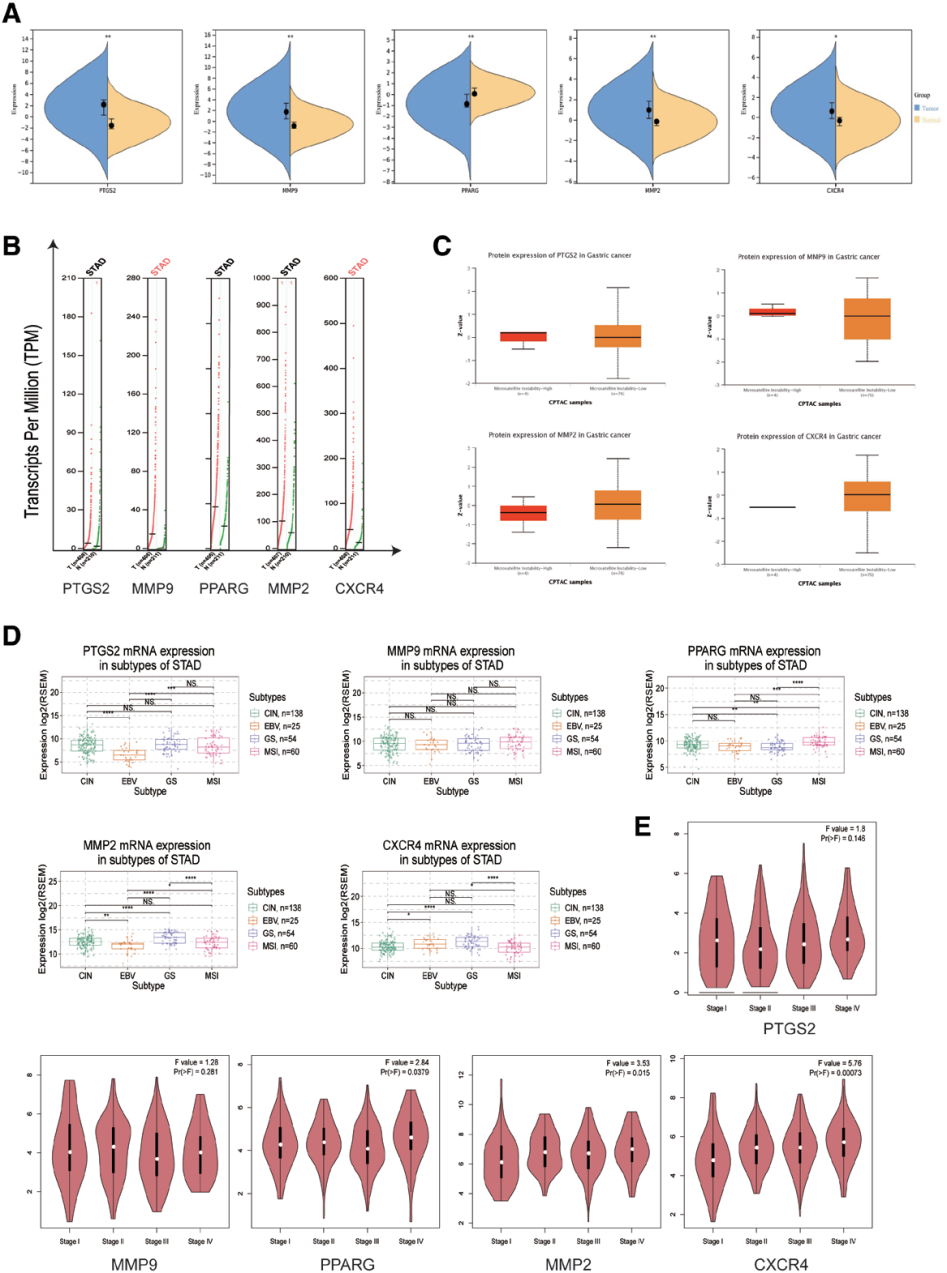
Clinical correlation analysis of the hub genes. (A) Differential expression of hub genes in normal gastric tissues and gastric cancer tissues. (B) Copy number analysis. (C) Protein expression of the hub genes. (D) The mRNA expression differences of hub genes in 4 subtypes of gastric cancer molecules, including Chromosomal Instability (CIN), Epstein-Barr virus (EBV), Genomically Stable (GS), and Microsatellite Instability (MSI). (E)Differential expression of the hub genes in different clinical stages.

The differences in the expression of hub genes (Fig. 6A), PTGS2, MMP9, MMP2, and CXCR4 genes were higher than normal gastric tissues in gastric cancer, and PPARG gene was lower than normal gastric tissues. MMP9 and CXCR4 were upregulated in gastric cancer in copy number analysis (Fig. 6B). In addition, the protein expression of PTGS2, MMP9, MMP2, and CXCR4 genes were significantly altered (Fig. 6C), which also corroborated the gene expression differences. To further optimize the precision treatment of GC, we analyzed the mRNA expression of hub genes in 4 subtypes, including Chromosomal Instability,^[54]^ Epstein-Barr virus,^[55]^ Genomically Stable (GS), and Microsatellite Instability (MSI),^[56]^ of which all 4 genes had different degrees of expression differences except for MMP9 (Fig. 6D). Then we analyzed the expression of hub genes across clinical stages (Fig. 6E), PPARG (*P < .05*), MMP2 (*P < .05*), and CXCR4 (*P < .001*), and the expression levels changed significantly with the advancement of pathological stages, indicating that the expression of the above genes causes obvious abnormal changes in cancer cells and tissues, and at the same time affects the tumor growth status and proliferation rate.

### 3.6. Pathologic findings and prognostic analysis of the hub genes

**Figure 7.**
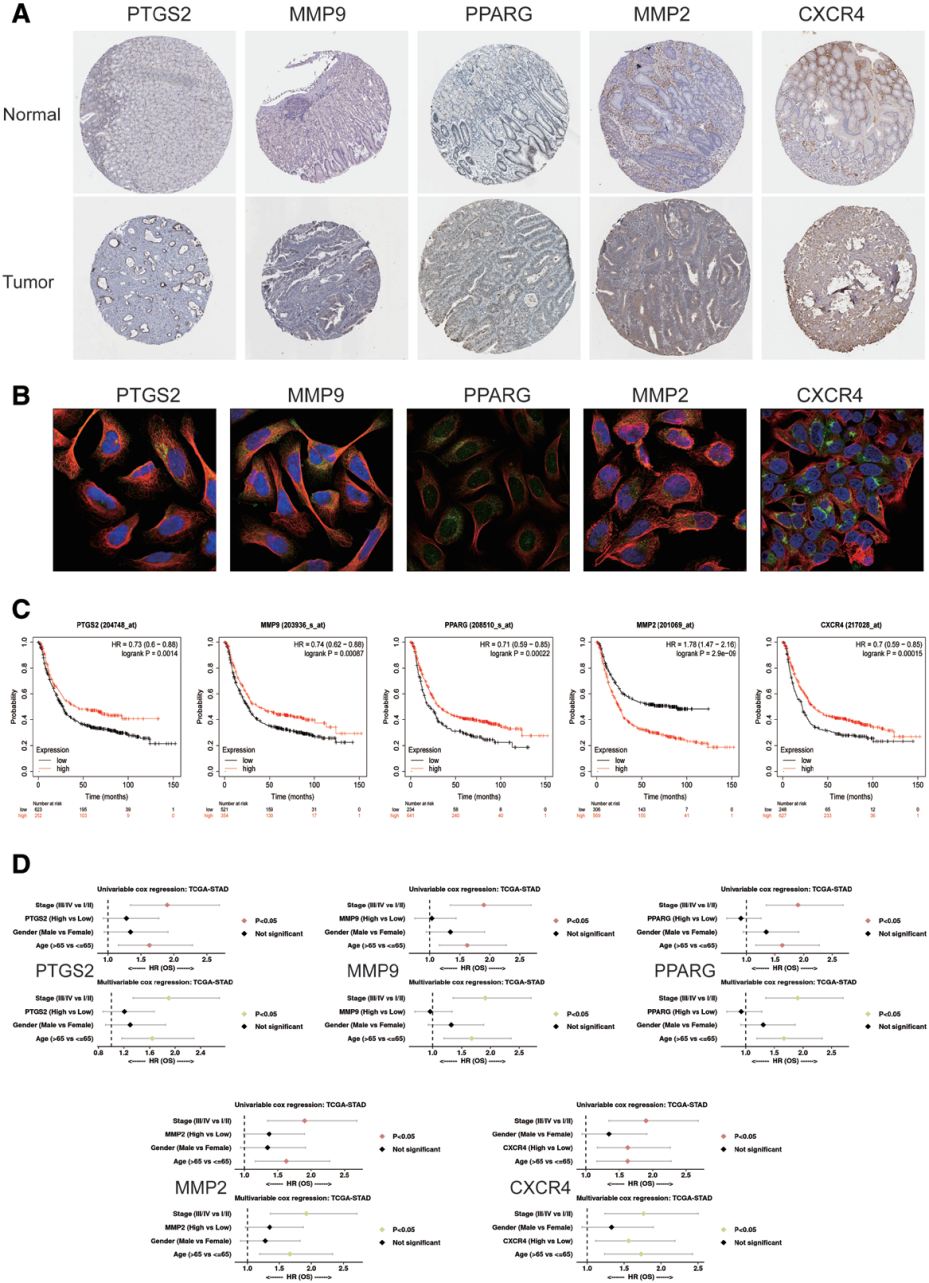
Pathologic findings and prognostic analysis of the hub genes. (A) Immunohistochemical pictures of gastric cancer and normal gastric glandular tissue. (B) Immunofluorescence images of the hub genes. (C) Correlation of hub genes expression with overall patient survival. (D) Hub genes expression in univariate and multivariate COX analysis. COX = cox proportional-hazards model.

The morphological changes of hub genes in gastric cancer and normal gastric glandular tissues were initially observed from the immunohistochemical pictures (Fig. 7A). Analysis from the acquired immunofluorescence pictures (Fig. 7B) revealed that PTGS2, MMP9, MMP2, and CXCR 4 were mostly located around the nucleus in tumor tissues, and PPARG was mostly located inside the nucleus in tumor tissues. Analyzing the correlation between hub genes expression and patients’ OS (Fig. 7C), PTGS2, MMP9, PPARG, and CXCR4 were positively correlated with patients’ OS, whereas those of MMP2 were negatively correlated with patients’ OS (*P < .01*). In both univariate and multivariate COX analyses (Fig. 7D), all 5 genes were associated with tumor stage and age (*P < .05*), which shows that gene mutation is an important cause of cancer development, and the tumor stage and age of the patient are one of the important factors.

### 3.7. Analysis of hub genes mutations

**Figure 8.**
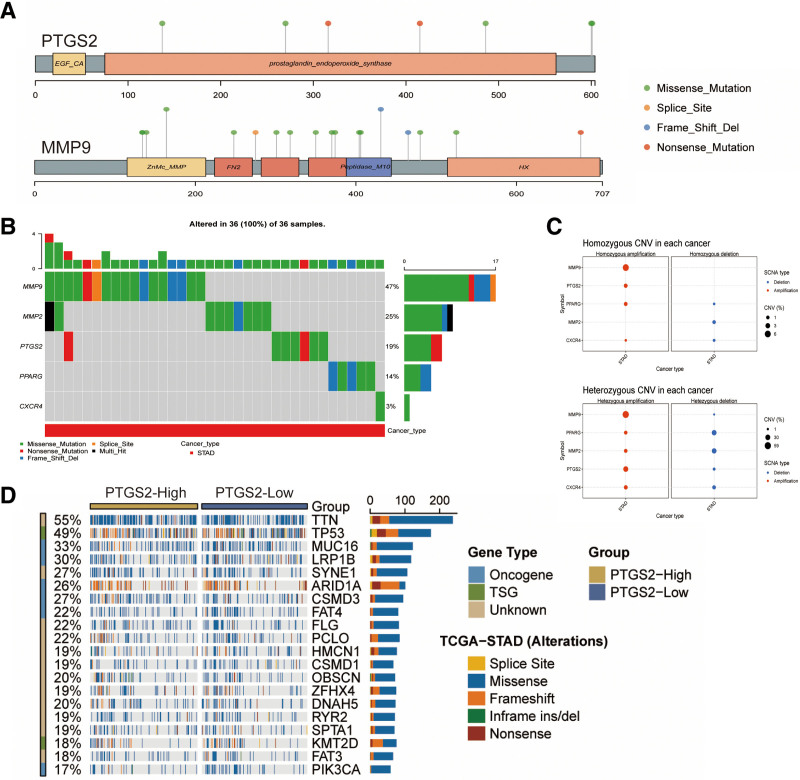
Analysis of Hub genes mutations. (A) Single nucleotide mutation (SNV) in the hub genes. (B) Mutation types and percentage of mutations in hub genes. (C) Hub genes copy number mutation (CNV). (D) The first ranked gene, PTGS2 gene, is associated with the top 20 Driver Mutation genes, including Oncogene, TSG, and Unknown classified genes.

We analyzed the mutation types of hub genes and the effect of mutations on the survival of GC patients. In SNV analysis, the mutation rates of PTGS2 and MMP9 were high (Fig. 8A). There were more MMP9 mutation sites (Fig. 8B), which were mainly dominated by missense mutation, followed by MMP2, and the least mutated was CXCR4. In CNV analysis (Fig. 8C), the MMP2 mutation type was mainly dominated by heterozygous deletion, whereas MMP9 and PTGS2 were mainly dominated by heterozygous amplification. In addition, the high or low expression of PTGS2 was most closely related to the TTN gene family, followed by TP53, and the type of Missense mutation was critical for its effect (Fig. 8D).^[57]^

### 3.8. Methylation of hub genes

**Figure 9.**
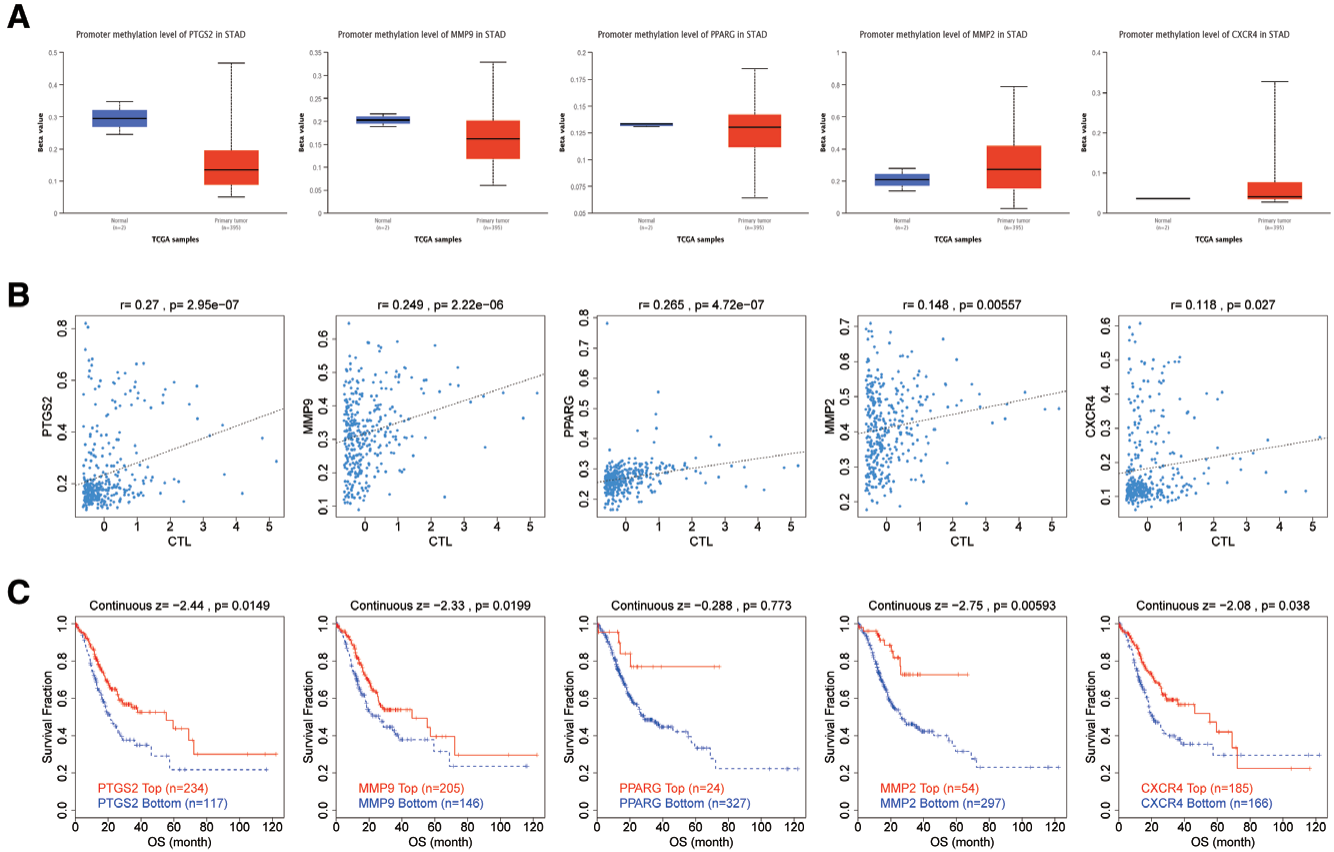
Methylation analysis of the Hub genes. (A) Expression of methylation levels of hub genes in normal and cancer tissues to analyze the relationship between hub genes and cancer methylation. (B) Correlation of hub genes methylation with cytotoxic T lymphocytes (CTL). (C) Relationship between hypermethylated and hypomethylated groups and prognostic survival in gastric cancer.

Analyzing the relationship between hub genes and disease methylation, we found that hub genes could affect the promoter methylation level, and the methylation level of hub genes was increased to different degrees in GC, with the most significant changes in PTGS2, which ranked first (Fig. 9A). After that, the correlation between hub genes methylation and CTL was analyzed, and it was found that the correlation was positive with *P < .05* and *R > 0*,^[58]^ indicating that CTL had a positive effect on the methylation of both central genes, and the correlation of PTGS2 was significant (Fig. 9B). In addition, analyzing the relationship between the hypermethylation and hypomethylation groups and the prognostic survival of gastric cancer, we found that PTGS2 (*P < .05*), MMP9 (*P < .05*), MMP2 (*P < .01*), and CXCR4 (*P < .05*), which were of significance for prognostic analyses, showed that the survival rate of the hypomethylation group was higher (Fig. 9C). It can be seen that regulating the hub genes and reducing the methylation level is beneficial to the prognosis of GC patients.

### 3.9. Immune infiltration and immune checkpoints

**Figure 10.**
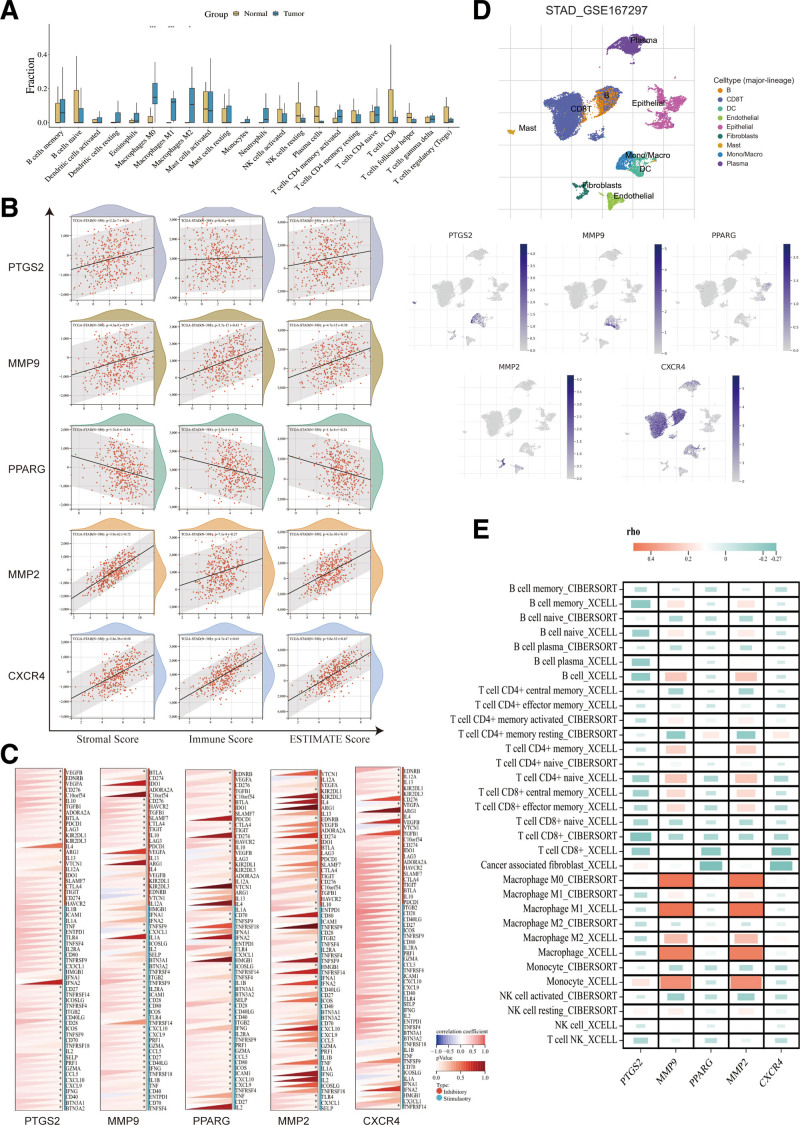
Immune infiltration and immune checkpoint analysis. (A) Immune infiltration analysis. (B) Correlation between hub genes and tumor immunity scores. (C) Correlation between the hub genes and tumor immune checkpoints. (D) The hub genes present in various immune cells. (E) Heatmap showing the correlation of hub genes with major immune cells in gastric cancer.

The results of immune infiltration showed (Fig. 10A) that GC was statistically highly expressed in macrophages (*P < .05*), suggesting that macrophage proliferation is a risk factor for GC. In Immune Score (Fig. 10B), except PTGS2, the rest of hub genes were statistically significant with immune checkpoints (*P < .05*).^[59]^ We further analyzed the hub genes and tumor immune checkpoints (Fig. 10C), which showed that all hub genes were statistically associated with immunosuppression (*P < .05*). In addition, single-cell analysis of the tumor microenvironment showed that both hub genes could be present in various immune cells (Fig. 10D). We demonstrated the correlation of hub genes with the major immune cells in GC (Fig. 10E), in which MMP9 and MMP2 mostly showed positive correlation (*R > 0*) in macrophages and monocytes, and the rest mostly negative correlation (*R < 0*).

### 3.10. Molecular docking

**Figure 11.**
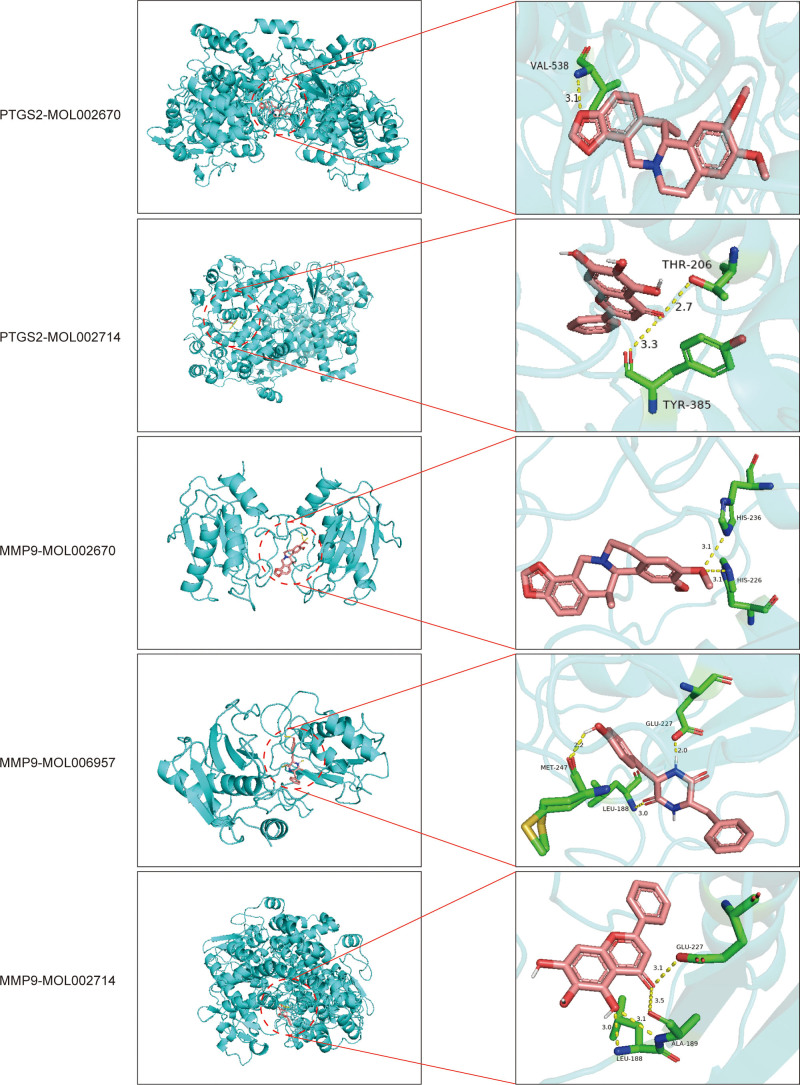
Visualize the top 5 results with the lowest binding energy.

The 5 key components were molecularly docked to each of the 5 core targets, and the binding energies were tabulated (Table 2). All docking binding energies were less than -5.0 kcal/mol, indicating that there was good binding capacity between Banxia-Shengjiang drug pair and the 5 core targets.^[60]^ The top 5 results with the lowest docking energies were finally selected for visualization and labeled with the crystal structure names and docking energy sizes of the hub genes (Fig. 11), among which MMP9 and PTGS2 had the relatively lowest binding energies to the components, which suggests to us that MMP9 and PTGS2 may be the main target genes for the anti-gastric cancer effect exerted by Banxia-Shengjiang drug pair.

**Table 2:** Molecular docking binding energy.

Source	MOI ID	Active Ingredients	Binding Affinity(kcal/mol)				
			PTGS2	MMP9	PPARG	MMP2	CXCR4
			(5F19)	(4WZV)	(6C5Q)	(8H78)	(3ODU)
Banxia	MOL002670	Cavidine	−9.5	−10.3	−9.0	−8.0	−8.9
Banxia	MOL006957	(3S,6S)-3-(benzyl)-6-(4-hydroxybenzyl)piperazine-2,5-quinone	−9.0	−10.4	−8.4	−8.9	−8.1
Banxia	MOL002714	baicalein	−9.3	−10.3	−7.6	−8.1	−8.3
Shengjiang	MOL006129	6-methylgingediacetate2	−7.4	−8.2	−6.7	−7.1	−6.2
Shengjiang	MOL008698	Dihydrocapsaicin	−7.4	−8.5	−6.4	−5.8	−6.4

CXCR4 = C-X-C motif chemokine receptor 4, MMP2 = matrix metalloproteinase-2, MMP9 = matrix metalloproteinase-9, PPARG = peroxisome, PTGS2 = prostaglandin-endoperoxide synthase 2 proliferator activated receptor gamma.

## 4. Discussion

We explored the potential targets of Banxia-Shengjiang drug pair to inhibit GC by network pharmacology, bioinformatics technology, and analyzed the correlation between the hub genes and immune infiltration, immunotherapy, and gene mutation, so as to provide theoretical basis for the treatment of GC by Banxia-Shengjiang drug pair in the clinic. First, the PPI network constructed from the intersecting target genes of Banxia-Shengjiang drug pair therapy for GC identified PTGS2, MMP9, PPARG, MMP2, and CXCR4 as the hub genes anti-GC. The major signaling pathways are associated with the RAS signaling pathway and the MAPK signaling pathway. Secondly, according to the results of clinical correlation analysis, the expression of PTGS2, MMP9, MMP2 and CXCR4 genes in gastric cancer is higher than that in normal gastric tissues, and the expression of PPARG gene in gastric cancer is lower.^[61–65]^ This indicates that the expression of the above genes causes obvious abnormal changes in cancer cells and tissues, and affects the growth status and proliferation rate of tumors. Furthermore, the correlation between hub genes and immune infiltration, gene mutation and methylation repair system will be analyzed to provide theoretical support for further precision treatment. Finally, the immune checkpoints and immune expression of hub genes confirmed that hub genes have therapeutic effects on gastric cancer through various different pathways. Meanwhile, molecular docking results also showed good binding ability between the core targets of both Banxia-Shengjiang drug pair and hub. Therefore, we concluded that Banxia-Shengjiang drug pair might exert therapeutic effects on gastric cancer by modulating the RAS/MAPK/MMP signaling pathway.

According to the analysis of the above results, the effect and mechanism of Banxia-Shengjiang drug pair in the treatment of gastric cancer are closely related to the RAS/MAPK/MMP signaling pathway. Studies in this area have shown that activation of the RAS/MAPK signaling pathway promotes the proliferation and migration of cancer cells.^[66,67]^ Therefore, by inhibiting RAS/MAPK signaling, carcinogenesis and progression can be inhibited. Matrix metalloproteinase (MMP) is considered to be an important component of tumor invasion in gastric cancer and a useful marker used to determine the biological aggressiveness of gastric cancer, and inhibition of the expression of these genes has a tumor-suppressive effect.^[68,69]^ On the other hand, MMPs were found to be highly correlated with the microenvironment of tumors and immune cells, and Youqiong Ye et al^[70]^ found that MMP2 and MMP9 regulate tumor immune checkpoints by modulating PD - L1. Liu Bo et al^[71]^ also found immunolocalization of MMP9 and MMP2 in osteolytic metastasis of human breast cancer cells. This highlights the increasingly important role of immunotherapy in the fight against cancer.^[72]^ In summary, we believe that inhibition of the RAS/MAPK/MMP pathway may inhibit the onset and development of gastric cancer, while having an immunomodulatory effect on the tumor microenvironment.

Similar studies have found that the Banxia-Shengjiang drug pair, the key components and its classical formula composition can significantly inhibit the RAS/MAPK/MMP signaling pathway, which in turn exerts anti-tumor effects.^[73–75]^ Shuai Yan et al^[76]^ found that the Banxia Xiexin Decoction had a significant tumor-suppressive effect on transplanted tumors of nude mice with colon cancer by regulating the MAPK signaling pathway, thus achieving the therapeutic effect of colon cancer. Hairunisa Indah et al^[77]^ found that Shengjiang extract could achieve breast cancer inhibition by acting on ERK2. In addition, in terms of immunotherapy, Feng Xiao-Yi et al^[78]^ found that a medicinal pair containing Banxia could play a role in regulating the tumor immune microenvironment by inhibiting the AKT/ STAT3/ ERK signaling pathway. Further, through clinical observation, it is known that Xiao-Ban-Xia Decoction plays an obvious therapeutic effect in nausea, vomiting and other symptoms of cancer patients, and improves the quality of life of cancer pain patients.^[79]^ In conclusion, consistent with the results of network pharmacological prediction, we believe that the Banxia-Shengjiang drug pair can inhibit gastric cancer, and its action is related to the RAS/MAPK signaling pathway, in which immunotherapy plays a crucial role, thus providing theoretical support for us to hypothesize that Banxia-Shengjiang drug pair exerts an anti-GC effect.

However, due to the problem of time and energy, we only conducted a theoretical study on the treatment of gastric cancer with Banxia-Shengjiang drug pair. There are still some limitations of our study. First, we need more experimental and clinical validation to support our future research. Second, the sample size of DEGs associated with GC is small and needs to be further enhanced in the future. Therefore, we need further experimental validation and clinical studies to confirm the reliability of these results and the feasibility of clinical application. In our future research, we will start from the following points. 1. We will study the therapeutic effect of Banxia-Shengjiang drug pair on GC in in vitro experiments and clinical validation. 2. We will further investigate the molecular mechanism of Banxia-Shengjiang drug pair in the treatment of GC through the methods of gene silencing, CO-IP, and EMSA.

## 5. Conclusion

In this study, a total of 5 targets and 5 key active ingredients were screened for the inhibition of gastric cancer by Banxia-Shengjiang drug pair. Our study demonstrated that Banxia-Shengjiang drug pair is closely related to gastric cancer, regulating the expression of core target proteins PTGS2, MMP9, PPARG, MMP2, and CXCR4 through the RAS/MAPK/MMP signaling pathway and improving the immune function of patients with gastric cancer, thus exerting an anti-gastric cancer effect.

## Acknowledgments

We sincerely thank the public databases mentioned in this article for generously sharing a large amount of data. We also thank Ms. Joanna for reviewing and polishing the manuscript.

## Author contributions

**Conceptualization:** Yi Nan.

**Data curation:** Yating Yang, Ling Yuan, Wenjing Liu, Doudou Lu.

**Formal analysis:** Yating Yang, Ling Yuan, Wenjing Liu, Doudou Lu, Fandi Meng.

**Funding acquisition:** Ling Yuan, Yi Nan.

**Investigation:** Yating Yang, Wenjing Liu, Fandi Meng.

**Methodology:** Yating Yang, Yi Yang.

**Resources:** Fandi Meng, Yi Yang.

**Software:** Ziying Zhou, Ping Ma.

**Writing – original draft:** Yating Yang, Ling Yuan.

**Writing – review & editing:** Yi Nan.

## Supplementary Material


